# CURRENT EFFORTS TO UNDERSTAND AND IMPROVE OLDER ADULTS’ FUNCTIONAL RECOVERY AFTER HIP FRACTURE

**DOI:** 10.1093/geroni/igz038.1564

**Published:** 2019-11-08

**Authors:** Richard H Fortinsky, Nancy Latham

**Affiliations:** 1 University of Connecticut School of Medicine, Farmington, Connecticut, United States; 2 Brigham and Women’s Hospital, Boston, Massachusetts, United States

## Abstract

The annual number of hip fractures in the US is projected to increase from 258,000 in 2010 to 458,000 by 2050. Globally, annual hip fractures are projected to reach 4.5 million by 2050. Yet the majority of older adults experience long-term mobility disability following hip fracture and do not return to pre-fracture functional capacity. Published reviews have concluded there is insufficient evidence regarding effectiveness of interventions designed to reduce residual disability and enhance mobility post-hip fracture. This Symposium features current efforts to understand and improve functional recovery post-hip fracture. Dr. Magaziner will present results from the recently-completed Community Ambulation Project (CAP), a multi-site randomized trial in which two in-home physical therapy interventions were compared: PUSH, which included aerobic conditioning, strengthening, balance and functional training; and PULSE, which included transcutaneous electrical nerve stimulation, flexibility and active range of motion exercises. Dr. Fortinsky will present CAP data examining the role of psychological resilience, optimism, depression, and balance confidence on mobility measures. Dr. Gruber-Baldini will present CAP data examining differences between PUSH and PULSE on study participants’ cognition and the impact of cognition on community ambulation. Dr. Binder will present the study design and early results from the STEP-HI study, a multi-site randomized trial evaluating whether structured exercise and topical testosterone therapy can improve function post-hip fracture among older women. Discussant Dr. Latham will comment on design, results, and implications of these two studies for research, policy, and practice intended to improve older adults’ recovery after hip fracture.

